# Cost-effectiveness of Atezolizumab Combination Therapy for First-Line Treatment of Metastatic Nonsquamous Non–Small Cell Lung Cancer in the United States

**DOI:** 10.1001/jamanetworkopen.2019.11952

**Published:** 2019-09-25

**Authors:** Steven D. Criss, Meghan J. Mooradian, Tina R. Watson, Justin F. Gainor, Kerry L. Reynolds, Chung Yin Kong

**Affiliations:** 1 Institute for Technology Assessment, Massachusetts General Hospital, Boston; 2 Massachusetts General Hospital Cancer Center, Boston; 3 Harvard Medical School, Boston, Massachusetts

## Abstract

**Importance:**

Immune checkpoint inhibitor combination therapy has recently become the standard of care for first-line treatment of metastatic nonsquamous non–small cell lung cancer. The implications of these first-line treatments are considerable, given the potential population of patients eligible to receive them and their high cost.

**Objective:**

To evaluate the cost-effectiveness of adding atezolizumab to bevacizumab, carboplatin, and paclitaxel as a first-line treatment strategy for patients with metastatic nonsquamous non–small cell lung cancer in the United States.

**Design, Setting, and Participants:**

In this economic evaluation, a primary microsimulation model was developed to assess atezolizumab combination vs bevacizumab, carboplatin, and paclitaxel alone in the first line (base case 1). A secondary model was developed to assess these treatments along with pembrolizumab combination and platinum doublet chemotherapy (base case 2). Treatment strategies and other simulated conditions were based on those from the IMpower150 and KEYNOTE-189 clinical trials. The study perspective was the US health care sector. One million patients with metastatic nonsquamous non–small cell lung cancer were simulated for each treatment group. This study was performed from February 2019 through May 2019.

**Main Outcomes and Measures:**

Incremental cost-effectiveness ratios were compared with a willingness-to-pay threshold of $100 000 per quality-adjusted life-year (QALY).

**Results:**

In base case 1, in which 1 million patients were simulated, treating with bevacizumab, carboplatin, and paclitaxel in the first line was associated with a mean cost of $112 551 (95% CI, $112 450-$112 653) and a mean survival of 1.48 QALYs (95% CI, 1.47-1.48 QALYs) per patient. Atezolizumab plus bevacizumab, carboplatin, and paclitaxel was associated with a mean cost of $244 166 (95% CI, $243 864-$244 468) and a mean survival of 2.13 QALYs (95% CI, 2.12-2.13 QALYs) per patient, for an estimated incremental cost-effectiveness ratio of $201 676 per QALY (95% CI, $198 105-$205 355 per QALY). In base case 2, in which 1 million patients were simulated, pembrolizumab combination therapy was associated with a mean cost of $226 282 (95% CI, $226 007-$226 557) and a mean survival of 2.45 QALYs (95% CI, 2.44-2.46 QALYs) per patient. Pembrolizumab combination dominated atezolizumab plus bevacizumab, carboplatin, and paclitaxel, leading to an incremental cost-effectiveness ratio of $116 698 per QALY (95% CI, $115 088-$118 342 per QALY) between pembrolizumab combination and bevacizumab, carboplatin, and paclitaxel. Atezolizumab combination was not cost-effective at a willingness-to-pay threshold of $100 000 per QALY.

**Conclusions and Relevance:**

In this simulated model economic analysis, atezolizumab combination was not cost-effective compared with bevacizumab, carboplatin, and paclitaxel and provided suboptimal incremental benefit compared with cost vs pembrolizumab combination for first-line treatment. Although atezolizumab combination therapy provides clinical benefits, price reductions may be necessary for this treatment strategy to become cost-effective.

## Key Points

**Question:**

Is atezolizumab plus bevacizumab, carboplatin, and paclitaxel (atezolizumab combination) cost-effective as a first-line treatment strategy for US patients with metastatic nonsquamous non–small cell lung cancer?

**Findings:**

In this economic evaluation of 1 million simulated patients with metastatic nonsquamous non–small cell lung cancer, adding atezolizumab to bevacizumab, carboplatin, and paclitaxel was not cost-effective at a willingness-to-pay threshold of $100 000 per quality-adjusted life-year, compared with bevacizumab, carboplatin, and paclitaxel alone. Atezolizumab combination also provided suboptimal incremental benefit compared with cost vs pembrolizumab combination for first-line treatment.

**Meaning:**

Although atezolizumab combination therapy provides clinical benefit, price reductions may be necessary for this treatment strategy to become cost-effective.

## Teaser

This economic evaluation examines whether atezolizumab plus bevacizumab, carboplatin, and paclitaxel is cost-effective vs bevacizumab, carboplatin, and paclitaxel alone as a first-line treatment for US patients with metastatic nonsquamous non–small cell lung cancer (NSCLC).

## Introduction

Immune checkpoint inhibitors (ICIs) have swiftly become the standard of care for patients with metastatic nonsquamous non–small cell lung cancer (NSCLC). For several years, ICIs were the therapy of choice for second-line treatment, with atezolizumab, pembrolizumab, and nivolumab—each a programmed death receptor 1 (PD-1) or programmed death ligand 1 (PD-L1) inhibitor—being the predominant options for patients with nonsquamous NSCLC who had been treated previously with chemotherapy.^1,2,3^ Given their success as second-line treatments, ICIs were subsequently used as first-line treatments with 2 strategies: ICI monotherapy and ICI in combination with chemotherapy.

Pembrolizumab monotherapy was the first of these PD-1 or PD-L1 ICIs to be approved for first-line use in October 2016 but was restricted to patients with metastatic NSCLC whose tumors had PD-L1 expression of at least 50% and no epithelial growth factor receptor or anaplastic lymphoma kinase mutations.^4^ In May 2017, the US Food and Drug Administration granted accelerated approval for pembrolizumab in combination with platinum and pemetrexed for patients with nonsquamous NSCLC without epithelial growth factor receptor or anaplastic lymphoma kinase mutations,^5^ with regular approval following in August 2018. Shortly thereafter in December 2018, atezolizumab became the second PD-1 and PD-L1 ICI approved for first-line treatment.^5,6^ Atezolizumab plus bevacizumab, carboplatin, and paclitaxel (ABCP) was shown to reduce mortality risk compared with bevacizumab, carboplatin, and paclitaxel (BCP) alone in the IMpower150 (ClinicalTrials.gov identifier, NCT02366143) clinical trial (hazard ratio for death, 0.78; 95% CI, 0.64-0.96; *P* = .02),^7^ while also significantly extending progression-free survival (PFS) compared with BCP (median, 8.3 months vs 6.8 months). On the basis of these data, patients with metastatic nonsquamous NSCLC without epithelial growth factor receptor or anaplastic lymphoma kinase mutations are now eligible to receive atezolizumab in combination with BCP.

The implications of PD-1 or PD-L1 ICI approval in the first-line treatment of nonsquamous NSCLC are considerable, given the potential population of patients eligible to receive these therapies and their high cost. Approximately 60% of 192 000 patients with NSCLC were estimated to receive a diagnosis of metastatic disease in 2018, and almost three-quarters of those patients were estimated to have a nonsquamous histologic type,^8,9^ leading to a newly eligible population of approximately 80 000 patients. Cost-effectiveness analysis can be an important tool to assess whether new therapies provide clinical benefit at a justifiable cost, which is increasingly necessary as approved indications continue to expand. Immune checkpoint inhibitors represent the fastest growing segment of the oncology therapeutics market, and more than 1000 trials testing PD-1 and PD-L1 ICIs are currently active,^10,11^ heightening the need for economic analyses of these therapies.

The cost-effectiveness of first-line pembrolizumab combination therapy has been studied previously and was estimated to be cost-effective at a willingness-to-pay (WTP) threshold of $180 000 per quality-adjusted life-year (QALY), with an incremental cost-effectiveness ratio (ICER) of $104 823 per QALY compared with chemotherapy.^12^ However, the most recently approved first-line ICI option—atezolizumab combination therapy—has yet to be investigated using cost-effectiveness analysis. The objective of our study was to evaluate the cost-effectiveness of adding atezolizumab to BCP as a first-line treatment strategy for US patients with metastatic nonsquamous NSCLC. In addition, we aim to evaluate the comparative cost-effectiveness of ABCP compared with pembrolizumab combination, the other prominent first-line ICI combination therapy.

## Methods

### Simulation Model

This study was exempted from institutional review board approval through Massachusetts General Hospital. Our study follows the Consolidated Health Economic Evaluation Reporting Standards (CHEERS) reporting guideline for economic evaluations.^13^

This study was performed from February 2019 through May 2019. Using TreeAge Pro statistical software version 2019 R1 (TreeAge Software), we developed a microsimulation model to estimate the health and cost outcomes of patients with metastatic nonsquamous NSCLC from the US health care sector perspective. Two treatment groups were included in the primary model (base case 1), on the basis of the IMpower150 clinical trial^7^: first-line treatment with either BCP only or ABCP. All patients entered the model in the PFS health state and could transition to progressive disease and death. A model cycle length of 1 month was chosen for the analysis. Health and cost outcomes were discounted at a 3% annual rate.^14^ To minimize the effect of statistical fluctuations on the outcomes, 1 million patients were simulated for each treatment group in the model. The ICER between the 2 groups was compared with a WTP threshold of $100 000 per QALY.^15^

**Figure 1.**
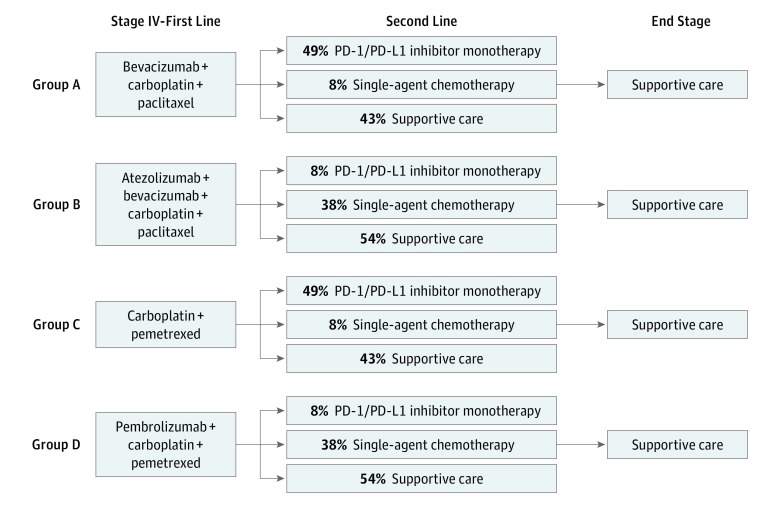
Summary Schematic of Treatment Strategies in Base Case 1 (Groups A and B) and Base Case 2 (Groups A, B, C, and D) Percentages refer to percentage of patients receiving each second-line option on the basis of subsequent therapy data from KEYNOTE-189.^16^ PD-1 indicates programmed death receptor 1; and PD-L1, programmed death ligand 1.

In a secondary analysis (base case 2), using TreeAge Pro 2019 R1, we performed an indirect treatment comparison between the treatment groups from the IMpower150 trial (ABCP vs BCP)^7^ and those from the KEYNOTE-189 trial (ClinicalTrials.gov identifier, NCT02578680; pembrolizumab plus platinum and pemetrexed vs platinum and pemetrexed alone).^16^ Although the IMpower150 and KEYNOTE-189 trials investigated treatment efficacy in 2 separately randomized patient populations, the similarity of the 2 sets of patient populations in terms of median age, ratio of female to male patients, Eastern Cooperative Oncology Group performance status scores, smoking status, and nonsquamous histologic subtypes should allow an exploratory comparison via cost-effectiveness analysis (eTable 1 in the Supplement). Four treatment groups were included in base case 2: first-line treatment with BCP only, ABCP, platinum and pemetrexed only, or pembrolizumab plus platinum and pemetrexed (pembrolizumab combination). The aforementioned modeling assumptions from base case 1 were also used in base case 2. Figure 1 summarizes the treatment lines for base case 1 and base case 2—groups A and B constitute base case 1, and groups A, B, C, and D constitute base case 2.

All patients received first-line treatment in the PFS health state. First-line treatment regimens and dosage in base case 1 followed those detailed by the IMpower150 trial.^7^ The regimens and dosage for the platinum plus pemetrexed and pembrolizumab combination options added in base case 2 followed those of the KEYNOTE-189 trial^16^ (platinum was modeled as carboplatin in both groups).

After progression, second-line therapy consisted of either PD-1 or PD-L1 monotherapy or additional chemotherapy. Subsequent therapy data were not published with the results of the IMpower150 trial,^7^ so we modeled patients as receiving types of second-line treatment similar to those detailed in the KEYNOTE-189 trial data.^16^ Because both trials combined ICIs with platinum-based chemotherapy in the first line, we assumed that the 2 populations would have similar tolerances for subsequent treatment.^7,16^ In base case 1 and base case 2, as in KEYNOTE-189,^16^ 56% of patients who progressed in the chemotherapy groups and 46% of patients who progressed in the ICI combination groups received subsequent therapy. Patients who did not receive subsequent therapy after progression were modeled as receiving only supportive care. eTable 2 in the Supplement provides additional information on each treatment regimen.

### Survival and Health State Utilities

Because the survival curves of patients receiving immunotherapy have been observed not to follow simple decay functions, Kaplan-Meier (KM) estimates from the clinical trials were first used in modeling PFS and overall survival (OS) projections. Progression-free survival projections were modeled using KM estimates for the first 6 months,^7,16^ followed by exponential functions fitted to the KM survival curves. Overall survival projections were modeled using KM estimates for the first year,^7,16^ then exponential functions were fitted to the KM survival curves through the fifth year, and mortality rates were estimated from Surveillance, Epidemiology, and End Results data^17,18^ thereafter. Surveillance, Epidemiology, and End Results estimates of mortality rates were derived from survival data from 2000 to 2014 for patients with metastatic nonsquamous NSCLC in the Surveillance, Epidemiology, and End Results database^17,18^ starting 2 months after diagnosis (eTable 3 in the Supplement), similar to patients in KEYNOTE-189 at baseline.^12^

For all model groups, we used a time-to-death approach in applying health state utilities to projected life-years. Quality-of-life results were not published along with the outcomes of the IMpower150 trial.^7^ Therefore, we assumed that utility values would be similar to those from the KEYNOTE-189 trial,^12^ because both trials combined ICIs with platinum-based chemotherapy and had roughly similar toxicity profiles.^7,16^ Additional information on health state utilities is provided in eTable 4 in the Supplement.

### Costs

Costs associated with cancer treatment in our analysis included drug acquisition, therapy administration, treatment of major adverse events, follow-up scans, monthly supportive care, and death-associated costs.^19,20,21,22^ Unit drug costs are based on the Centers for Medicare & Medicaid Services April 2019 Average Sales Price Drug Pricing Files (version updated March 22, 2019).^19^ In calculating dosage amounts, body weight was assumed to be 70.32 kg (95% CI, 69.71-70.93 kg) and body surface area was assumed to be 1.79 m^2^ (95% CI, 1.78-1.80 m^2^), according to an analysis of an internal database containing data for more than 3500 patients with lung cancer treated at Partners Healthcare hospitals. Costs for treating adverse events that were rated as grade 3 or higher were considered in the model.^16,23,24^ Treatment cost estimates for these adverse events were sourced from the Agency for Healthcare Research and Quality’s Healthcare Cost and Utilization Project^21^ using corresponding *International Classification of Diseases*, *Ninth Revision* codes. Cost inputs used in the model are detailed in eTable 4 in the Supplement. Costs were adjusted to 2019 US dollars using the Personal Healthcare Price Index published by the Centers for Medicare & Medicaid Services.^25,26^

### Sensitivity Analysis

Using TreeAge Pro 2019 R1, 1-way deterministic sensitivity analyses were performed on key model variables to assess how uncertainty in these inputs could affect the cost-effectiveness results. Model variables were tested at the upper and lower limits of their respective 95% CIs or plausible ranges (eTable 5 in the Supplement). Sensitivity analyses were performed for both base case 1 and base case 2. In addition, to test the robustness of our findings when many model inputs are varied at once, we performed a probabilistic sensitivity analysis using 1000 iterations. Model inputs that were varied simultaneously in the probabilistic sensitivity analysis include those for health state utilities, costs, survival, body weight, and body surface area. Additional details on the probabilistic sensitivity analysis can be found in the eFigure in the Supplement.

## Results

### Incremental Cost-effectiveness Ratios

In base case 1, treating patients with metastatic nonsquamous NSCLC with BCP in the first line was associated with a mean cost of $112 551 (95% CI, $112 450-$112 653) and mean survival of 1.48 QALYs (95% CI, 1.47-1.48 QALYs) per patient. Adding atezolizumab to this BCP regimen was associated with a mean cost of $244 166 (95% CI, $243 864-$244 468) and mean survival of 2.13 QALYs (95% CI, 2.12-2.13 QALYs) per patient, for an estimated ICER of $201 676 per QALY (95% CI, $198 105-$205 355 per QALY) (Table 1).

**Table 1.** Summary Base Case 1 Results

Outcome	Bevacizumab Plus Carboplatin Plus Paclitaxel	Atezolizumab Plus Bevacizumab Plus Carboplatin Plus Paclitaxel
Cost, mean (95% CI), $US	112 551 (112 450-112 653)	244 166 (243 864-244 468)
QALY, mean (95% CI)	1.48 (1.47-1.48)	2.13 (2.12-2.13)
Incremental cost, $US		131 615
Incremental QALY		0.65
Incremental cost-effectiveness ratio per QALY, mean (95% CI), $US		201 676 (198 105-205 355)

Abbreviation: QALY, quality-adjusted life-year.

In base case 2, the carboplatin plus pemetrexed treatment strategy produced a mean cost of $82 738 (95% CI, $82 664-$82 812) and mean survival of 1.11 QALYs (95% CI, 1.10-1.11 QALYs) per patient. The ICER between BCP and carboplatin plus pemetrexed was, therefore, estimated to be $80 671 per QALY (95% CI, $78 640-$82 785 per QALY) (Table 2). Treating patients with pembrolizumab combination therapy was associated with a mean cost of $226 282 (95% CI, $226 007-$226 557) and mean survival of 2.45 QALYs (95% CI, 2.44-2.46 QALYs) per patient. Because pembrolizumab combination therapy had greater incremental QALYs and lower incremental cost compared with BCP than did ABCP, the pembrolizumab combination strategy dominated the ABCP treatment strategy. When including only nondominated strategies, compared with BCP, pembrolizumab combination was associated with an ICER of $116 698 per QALY (95% CI, $115 088-$118 342 per QALY) (Table 2). Comparing pembrolizumab combination with carboplatin plus pemetrexed using the cost and QALY outputs from our model would yield an ICER of $106 792 per QALY (95% CI, $105 779-$107 820 per QALY).

**Table 2.** Summary Base Case 2 Results

Outcome	Carboplatin Plus Pemetrexed	Bevacizumab Plus Carboplatin Plus Paclitaxel	Pembrolizumab Plus Carboplatin Plus Pemetrexed	Atezolizumab Plus Bevacizumab Plus Carboplatin Plus Paclitaxel
Cost, mean (95% CI), $US	82 738 (82 664-82 812)	112 551 (112 450-112 653)	226 282 (226 007-226 557)	244 166 (243 864-244 468)
QALY, mean (95% CI)	1.11 (1.10-1.11)	1.48 (1.47-1.48)	2.45 (2.44-2.46)	2.13 (2.12-2.13)
Incremental cost, $US		29 814	113 731	
Incremental QALY		0.37	0.97	Dominated
Incremental cost-effectiveness ratio per QALY, mean (95% CI), $US		80 671 (78 640-82 785)	116 698 (115 088-118 342)	

Abbreviation: QALY, quality-adjusted life-year.

### Sensitivity Analysis

**Figure 2.**
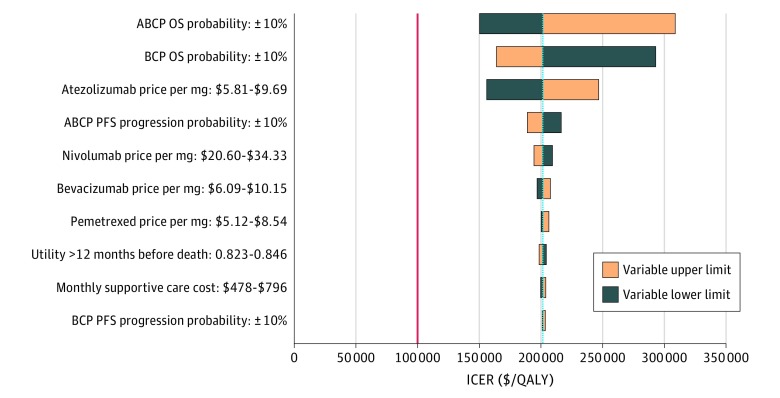
Deterministic Sensitivity Analysis for Base Case 1: Atezolizumab Plus Bevacizumab Plus Carboplatin Plus Paclitaxel (ABCP) vs Bevacizumab Plus Carboplatin Plus Paclitaxel (BCP) The red line signifies the $100 000 per quality-adjusted life-year (QALY) willingness-to-pay threshold used in this study, and the blue line presents the base case values. The ranges for each variable listed signify the lower and upper bounds used in the sensitivity analysis. The top 10 variables by magnitude of effect are shown. ICER indicates incremental cost-effectiveness ratio; OS overall survival; and PFS, progression-free survival.

Atezolizumab plus bevacizumab, carboplatin, and paclitaxel was not cost-effective at any of the tested variable lower or upper limits compared with BCP (Figure 2). Large decreases in the ICER between ABCP and BCP occurred at the lower limits of ABCP OS probability ($150 183 per QALY) and atezolizumab price ($155 949 per QALY), as well as the upper limit of BCP OS probability ($163 785 per QALY). Other variables had minimal effects on the ICER for ABCP vs BCP (the total ICER range between lower and upper limits was less than $30 000 per QALY).

**Figure 3.**
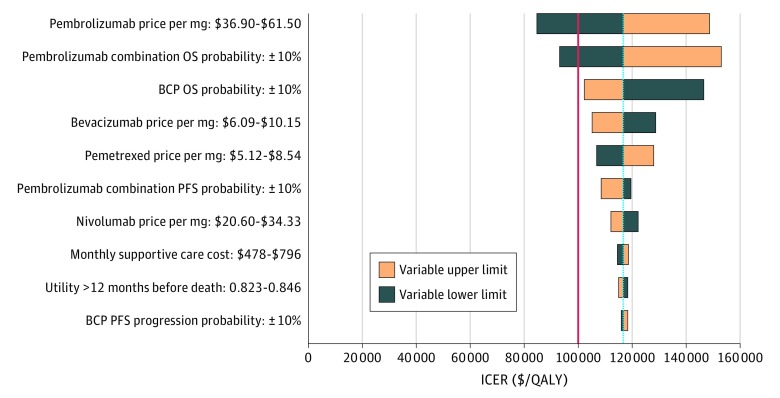
Deterministic Sensitivity Analysis for Base Case 2: Pembrolizumab Combination vs Bevacizumab Plus Carboplatin Plus Paclitaxel (BCP) The red line signifies the $100 000 per quality-adjusted life-year (QALY) willingness-to-pay threshold used in this study, and the blue line presents the base case values. The ranges for each variable listed signify the lower and upper bounds used in the sensitivity analysis. The top 10 variables by magnitude of effect are shown. ICER indicates incremental cost-effectiveness ratio; OS overall survival; and PFS, progression-free survival.

When testing base case 2, pembrolizumab combination was cost-effective at the lower limits of both the pembrolizumab price ($84 550 per QALY) and pembrolizumab combination OS probability ($92 928 per QALY) variables when compared with BCP (Figure 3). The upper limits of BCP OS probability and bevacizumab price led to ICERs near the WTP threshold ($102 258 per QALY and $105 099 per QALY, respectively). Atezolizumab plus bevacizumab, carboplatin, and paclitaxel was either dominated or weakly dominated in each of the tested variable limits. Therefore, ABCP did not appear on the efficient frontier, which represents the successive strategies that provide the greatest incremental effectiveness per incremental cost (the ICER being the inverse of the slope between 2 points on the frontier), in any case.

In performing the probabilistic sensitivity analysis for the base case 1 model, compared with BCP, ABCP produced an ICER greater than the WTP threshold of $100 000 per QALY in 100% of the 1000 iterations. The WTP threshold at which ABCP had a 50% probability of being cost-effective was approximately $205 000 per QALY. A 60% price reduction would allow ABCP to reach an ICER below the WTP threshold. When simultaneously varying the model inputs for the base case 2 model, compared with BCP, pembrolizumab combination yielded an ICER greater than the WTP threshold in 90% of the iterations. Pembrolizumab combination had a 50% probability of being cost-effective at a WTP threshold of approximately $125 000 per QALY.

## Discussion

Using a microsimulation model, we compared first-line treatment strategies with ABCP and BCP in base case 1 and found that ABCP does not provide a cost-effective means of delivering first-line treatment for patients with metastatic nonsquamous NSCLC. In base case 2, which was meant to provide exploratory cost-effectiveness estimates for the broader set of immunotherapy combination options in the first line, we added study groups for pembrolizumab combination and carboplatin plus pemetrexed. We found that BCP is cost-effective compared with carboplatin plus pemetrexed and that ABCP is dominated by pembrolizumab combination, although pembrolizumab combination remained above the cost-effectiveness WTP threshold compared with BCP.

Although ABCP has been shown to reduce overall mortality risk compared with BCP (hazard ratio for death, 0.78; 95% CI, 0.64-0.96; *P* = .02),^7^ on the basis of our results and the WTP threshold used in our study, its cost is not commensurate with the improvement it can provide. The most realistic action that can be taken to move ABCP toward cost-effectiveness would be a reduction in the price of atezolizumab. We found that a 60% price reduction would allow ABCP to reach an ICER below the WTP threshold. Because OS probabilities were the only other variables with large effects on the ICER (Figure 2), changing the price of atezolizumab is the clearest actionable strategy for achieving efficient use of ABCP.

Our study indicates that the cost-effectiveness of ABCP is insufficient compared with other options for first-line treatment of metastatic nonsquamous NSCLC. In base case 2, pembrolizumab combination was shown to be the preferred option over ABCP because of its lower total cost and superior projected survival benefit. Although pembrolizumab combination was not estimated to be cost-effective compared with BCP, it was able to provide a more-efficient balance between incremental cost and quality-adjusted survival gained than was ABCP. There is uncertainty regarding the base case 2 results, because we aggregated clinical trial results from 2 separately randomized patient populations. However, given that a head-to-head trial of ABCP and pembrolizumab combination is unlikely, our exploratory approach to comparing these 2 ICI combination therapies aims to advance the discussion around how these treatment strategies are used in the first line.

A previous analysis studied the cost-effectiveness of pembrolizumab combination and reported an ICER of $104 823 per QALY.^12^ However, our results cannot be compared directly, because the previous study evaluated pembrolizumab combination against chemotherapy alone,^12^ whereas, in our study, pembrolizumab combination was compared with the BCP group because of their respective rankings on the efficient frontier. Comparing pembrolizumab combination with carboplatin plus pemetrexed using the cost and QALY outputs from our model would yield an ICER of $106 792 per QALY (95% CI, $105 779-$107 820 per QALY). Although our ICER for pembrolizumab combination vs chemotherapy alone is similar to that of the previous study, our conclusions are different because they used a WTP threshold of $180 000 per QALY.^12^

Atezolizumab plus bevacizumab, carboplatin, and paclitaxel does provide clinical benefit in the first-line treatment of metastatic nonsquamous NSCLC and remains an approved option for patients. Progression-free survival was significantly longer for patients receiving ABCP across all PD-L1 expression levels, including those with less than 1% expression, and for patients with epithelial growth factor receptor or anaplastic lymphoma kinase mutations after the failure of treatment with tyrosine kinase inhibitor therapy—patient subgroups that are notably difficult to treat with single-agent ICI.^7^ Further research into whether OS can be improved in these patient subgroups is necessary, but preliminary results from the IMpower150 trial^7^ provide evidence that ABCP can fill an important role in treatment of metastatic nonsquamous NSCLC, particularly in certain patient populations. For these reasons, a price reduction to better align ABCP’s cost with its clinical benefit is all the more warranted.

### Limitations

Our study has several limitations. First, we assumed that subsequent therapy for patients in the ABCP and BCP groups would be comparable to those given to patients in the pembrolizumab combination and carboplatin plus pemetrexed groups, respectively. Subsequent therapy data were not published from the IMpower150 trial, but given that both trials combined ICIs with chemotherapy,^7,16^ it is likely that patients would be treated with similar types of regimens in the second line. Second, because the IMpower150 trial did not publish quality-of-life utility data, we assumed that the patients’ quality of life was similar to that of the patients in the KEYNOTE-189 trial.^12^ This assumption is based on the similarity of the treatment strategies and the approximate agreement of adverse event rates between trials,^7,16^ although the patients’ experiences could have been different between trials. Third, a low percentage of patients were modeled as having received single-agent ICI in the second line after receiving ABCP or pembrolizumab combination, on the basis of subsequent therapy data from the KEYNOTE-189 trial.^16^ There is uncertainty regarding whether patients who have already received an ICI in the first line should be given further treatment with a different ICI; however, we included this as an option for second-line treatment in our model to reflect a potential strategy for treating patients who are not well enough to receive chemotherapy and to emulate practice patterns at sites participating in pivotal clinical trials. Fourth, base case 2 combines results from 2 separately randomized patient populations, as mentioned earlier, which could bias the model’s outcomes if these patient populations were significantly different. However, this analysis should be viewed as a tentative estimate in the absence of a head-to-head trial of ABCP and pembrolizumab combination.

## Conclusions

Base case 1 estimated that ABCP would not be a cost-effective option at a WTP threshold of $100 000 per QALY. Pembrolizumab combination represented a better trade-off than ABCP between incremental cost and quality-adjusted survival in base case 2, although it was also not cost-effective at the WTP threshold. Price reductions remain the most pragmatic solution for the insufficient cost-effectiveness of these PD-1 or PD-L1 ICIs in the treatment of metastatic nonsquamous NSCLC.

## Notes

Supplement. **eTable 1. **Patient Characteristics Comparison: IMpower150 and KEYNOTE-189 **eTable 2. **Treatment Regimens **eTable 3. **SEER Survival Analysis: Stage IV Nonsquamous NSCLC (2000-2014) **eTable 4. **Cost and Utility Parameters **eTable 5. **Sensitivity Analysis Parameters **eFigure. **Acceptability Curves for Probabilistic Sensitivity Analysis **eReferences**
