# Supplementary material for: Cost-effectiveness of Atezolizumab Combination Therapy for First-Line Treatment of Metastatic Nonsquamous Non–Small Cell Lung Cancer in the United States
Source: JAMA Netw Open. 2019 Sep 25;2(9):e1911952. doi: 10.1001/jamanetworkopen.2019.11952 (PMC6764123; doi:10.1001/jamanetworkopen.2019.11952)
Supplement: Supplement. — eTable 1. Patient Characteristics Comparison: IMpower150 and KEYNOTE-189 eTable 2. Treatment Regimens eTable 3. SEER Survival Analysis: Stage IV Nonsquamous NSCLC (2000-2014) eTable 4. Cost and Utility Parameters eTable 5. Sensitivity Analysis Parameters eFigure. Acceptability Curves for Probabilistic Sensitivity Analysis eReferences [file jamanetwopen-2-e1911952-s001.pdf]

# Supplementary Online Content

Criss SD, Mooradian MJ, Watson TR, Gainor JF, Reynolds KL, Kong CY. Cost-effectiveness of atezolizumab combination therapy for first-line treatment of metastatic nonsquamous non–small cell lung cancer in the United States. *JAMA Netw Open*. 2019;2(9):e1911952. doi:10.1001/jamanetworkopen.2019.11952

**eTable 1.** Patient Characteristics Comparison: IMpower150 and KEYNOTE-189
**eTable 2.** Treatment Regimens
**eTable 3.** SEER Survival Analysis: Stage IV Nonsquamous NSCLC (2000-2014)
**eTable 4.** Cost and Utility Parameters
**eTable 5.** Sensitivity Analysis Parameters
**eFigure.** Acceptability Curves for Probabilistic Sensitivity Analysis
**eReferences**

This supplementary material has been provided by the authors to give readers additional information about their work.

© 2019 Criss SD et al. *JAMA Network Open.*

## eTable 1. Patient Characteristics Comparison: IMpower150 and KEYNOTE-189

| | IMpower150 [1] | | KEYNOTE-189 [2] | |
|---|---|---|---|---|
| **Characteristic** | **ABCP** | **BCP** | **Pembrolizumab Combination** | **Chemotherapy** |
| Median Age (range)—years | 63 (31-89) | 63 (31-90) | 65 (34-84) | 64 (34-84) |
| Male Sex | 60% | 60% | 62% | 53% |
| Eastern Cooperative Oncology Group Score = 0 or 1 | 100% | 100% | 100% | 100% |
| Current or Former Smoker | 80% | 81% | 88% | 88% |
| Adenocarcinoma Histology | 95% | 94% | 96% | 96% |

ABCP, atezolizumab + bevacizumab + carboplatin + paclitaxel; BCP, bevacizumab + carboplatin + paclitaxel

© 2019 Criss SD et al. *JAMA Network Open.*

## eTable 2. Treatment Regimens

| Variable | Input Value | Source |
|---|---|---|
| **Dose Sizes** | | |
| Bevacizumab + Carboplatin + Paclitaxel | bevacizumab, 15 mg/kg; carboplatin, AUC 6.0 mg/ml/min; paclitaxel, 200 mg/ $m^2$ | Socinski, et al, 2018 [1] |
| Atezolizumab + Bevacizumab + Carboplatin + Paclitaxel | atezolizumab, 1200 mg fixed bevacizumab, 15 mg/kg; carboplatin, AUC 6.0 mg/ml/min; paclitaxel, 200 mg/ $m^2$ | Socinski, et al, 2018 [1] |
| Pembrolizumab + Carboplatin + Pemetrexed | pembrolizumab, 200 mg fixed; carboplatin, AUC 6.0 mg/ml/min; pemetrexed, 500 mg/$m^2$ | Gandhi, et al., 2018 [2] |
| Carboplatin + Pemetrexed | carboplatin, AUC 6.0 mg/ml/min; pemetrexed, 500 mg/$m^2$ | Gandhi, et al., 2018 [2] |
| Pemetrexed (second line) | pemetrexed, 500 mg/$m^2$ | Gandhi, et al., 2018 [2] |
| Docetaxel (second line) | 75 mg/$m^2$ | Herbst, et al., 2016 [3] |
| Nivolumab (second line) | 480 mg fixed | Long, et al., 2018 [4] |
| **Infusion Timing** | | |
| Bevacizumab + Carboplatin + Paclitaxel | 3 weeks up to 4 cycles; bevacizumab only thereafter | Socinski, et al, 2018 [1] |
| Atezolizumab + Bevacizumab + Carboplatin + Paclitaxel | 3 weeks up to 4 cycles; atezolizumab and bevacizumab only thereafter | Socinski, et al, 2018 [1] |
| Pembrolizumab + Carboplatin + Pemetrexed | 3 weeks up to 4 cycles; pembrolizumab up to 35 cycles; pemetrexed only thereafter | Gandhi, et al., 2018 [2] |
| Carboplatin + Pemetrexed | 3 weeks up to 4 cycles; pemetrexed only thereafter | Gandhi, et al., 2018 [2] |
| Pemetrexed (second line) | 3 weeks | Gandhi, et al., 2018 [2] |
| Docetaxel (second line) | 3 weeks | Herbst, et al., 2016 [3] |
| Nivolumab (second line) | 4 weeks | Long, et al., 2018 [4] |

© 2019 Criss SD et al. *JAMA Network Open.*

**eTable 3. SEER Survival Analysis: Stage IV Nonsquamous NSCLC (2000-2014) [a]**

| Patient Summary Table | |
|---|---|
| Total patients in analysis (number) | 58,310 |
| **Observed Survival Estimates** | |
| 12 months | 38.20% |
| 24 months | 18.60% |
| 36 months | 11.20% |
| 48 months | 7.50% |
| 60 months | 5.50% |
| 72 months | 4.40% |
| 84 months | 3.60% |
| 96 months | 3.10% |
| 108 months | 2.70% |
| 120 months | 2.40% |
| 132 months | 2.10% |
| 144 months | 1.90% |
| 156 months | 1.80% |
| 168 months | 1.60% |
| 180 months | 1.60% |

[a] Analysis starts from two months post-diagnosis[5]

© 2019 Criss SD et al. *JAMA Network Open.*

## eTable 4. Cost and Utility Parameters

| Variable | Input Value | Source |
|---|---|---|
| **Costs (2019 USD)** | | |
| Atezolizumab price per mg | $7.75 | CMS 2019 ASP Drug Pricing Files [6] |
| Bevacizumab price per mg | $8.12 | CMS 2019 ASP Drug Pricing Files [6] |
| Pembrolizumab price per mg | $49.20 | CMS 2019 ASP Drug Pricing Files [6] |
| Pemetrexed price per mg | $6.83 | CMS 2019 ASP Drug Pricing Files [6] |
| Carboplatin price per mg | $0.06 | CMS 2019 ASP Drug Pricing Files [6] |
| Docetaxel price per mg | $1.16 | CMS 2019 ASP Drug Pricing Files [6] |
| Nivolumab price per mg | $27.46 | CMS 2019 ASP Drug Pricing Files [6] |
| Paclitaxel price per mg | $0.15 | CMS 2019 ASP Drug Pricing Files [6] |
| Imaging/Surveillance | $1,408.77 | CMS.gov [7], CPT 78816 |
| Drug administration per hour | $143.08 | CMS.gov [7], CPT 96413 |
| Monthly supportive care (regression estimate for 70 year old) | $637 | SEER-Medicare regression analysis |
| Death costs (regression estimate for 70 year old) | $9,433 | SEER-Medicare regression analysis |
| Adverse events (atezolizumab + bevacizumab + carboplatin + paclitaxel) | $5,204.17 | hcupnet.ahrq.gov; Socinski, et al, 2018 [1] |
| Adverse events (bevacizumab + carboplatin + paclitaxel) | $3,924.44 | hcupnet.ahrq.gov; Socinski, et al, 2018 [1] |
| Adverse events (pembrolizumab + carboplatin + pemetrexed) | $4,778.56 | hcupnet.ahrq.gov; Gandhi, et al., 2018 [2] |
| Adverse events (carboplatin + pemetrexed) | $3,911.34 | hcupnet.ahrq.gov; Gandhi, et al., 2018 [2] |
| Adverse events (second line) | $2,439.48 | hcupnet.ahrq.gov; Herbst, et al., 2016 [3] |
| **Utilities [a]** | | |
| >12 months prior to death | 0.834 | Insinga, et al., 2018 [5] |
| 6-12 months prior to death | 0.765 | Insinga, et al., 2018 [5] |
| 1-6 months prior to death | 0.709 | Insinga, et al., 2018 [5] |
| 1 month prior to death | 0.563 | Insinga, et al., 2018 [5] |

[a] Health utility values were sourced from Insinga, et al., 2018, a cost-effectiveness analysis performed by the Center for Observational and Real-World Evidence of Merck Sharp & Dohme Corp., the funding sponsor of the KEYNOTE-189 trial. Health utility values were not published as part of the KEYNOTE-189 trial results publication. Insinga, et al., 2018, notes that health utility values for the U.S. were calculated using EuroQOL-5D 3-level utility data collected from patients in KEYNOTE-189. Further details have been previously published in Insinga, et al., 2018.[5]

© 2019 Criss SD et al. *JAMA Network Open.*

## eTable 5. Sensitivity Analysis Parameters

| Variables | Mean | Lower | Upper |
|---|---|---|---|
| **Utilities** | | | |
| >12 months prior to death | 0.834 | 0.823 | 0.846 |
| 6-12 months prior to death | 0.765 | 0.743 | 0.786 |
| 1-6 months prior to death | 0.709 | 0.690 | 0.728 |
| 1 month prior to death | 0.563 | 0.461 | 0.665 |
| **Costs** | | | |
| Atezolizumab Price/mg [a] | $7.75 | $5.81 | $9.69 |
| Bevacizumab Price/mg [a] | $8.12 | $6.09 | $10.15 |
| Pembrolizumab Price/mg [a] | $49.20 | $36.90 | $61.50 |
| Pemetrexed Price/mg [a] | $6.83 | $5.12 | $8.54 |
| Carboplatin Price/mg [a] | $0.06 | $0.05 | $0.08 |
| Paclitaxel Price/mg [a] | $0.15 | $0.11 | $0.19 |
| Nivolumab Price/mg [a] | $27.46 | $20.60 | $34.33 |
| Docetaxel Price/mg [a] | $1.16 | $0.87 | $1.45 |
| Monthly Supportive Care Cost (regression estimate for 70 year old) [a] | $637 | $478 | $796 |
| Death Cost (regression estimate for 70 year old) [a] | $9,433 | $7,075 | $11,791 |
| **Survival** | | | |
| ABCP PFS progression probability | KM estimates, then 0.092 +/- 10% | | |
| BCP PFS progression probability | KM estimates, then 0.185 +/- 10% | | |
| Carboplatin + Pemetrexed PFS progression probability | KM estimates, then 0.140 +/- 10% | | |
| Pembrolizumab Combination PFS progression probability | KM estimates, then 0.081 +/- 10% | | |
| ABCP OS probability | KM estimates, then 0.034, then SEER +/- 10% | | |
| BCP OS probability | KM estimates, then 0.049, then SEER +/- 10% | | |
| Carboplatin + Pemetrexed OS probability | KM estimates, then 0.057, then SEER +/- 10% | | |
| Pembrolizumab Combination OS probability | KM estimates, then 0.029, then SEER +/- 10% | | |
| **Other** | | | |
| Body Weight (kilograms) | 70.32 | 69.71 | 70.93 |
| Body Surface Area ($meters^2$) | 1.79 | 1.78 | 1.80 |
| [a] range indicates 25% change | | | |
| Abbreviations: ABCP, atezolizumab + bevacizumab + carboplatin + paclitaxel; PFS, progression-free survival; BCP bevacizumab + carboplatin + paclitaxel; OS, overall survival; KM, Kaplan-Meier | | | |

© 2019 Criss SD et al. *JAMA Network Open.*

## eFigure. Acceptability Curves for Probabilistic Sensitivity Analysis

a) Atezolizumab plus BCP versus BCP

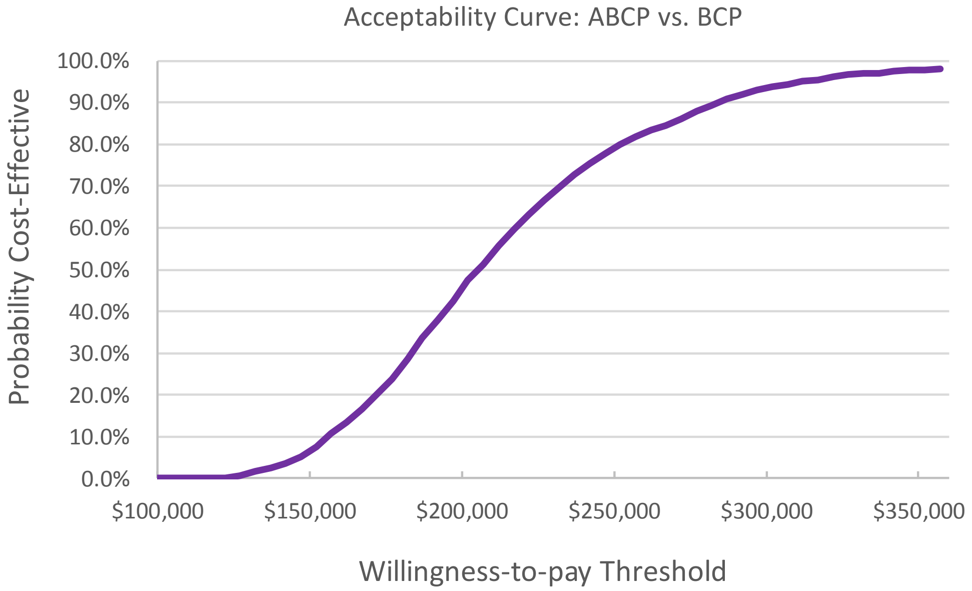


b) Pembrolizumab combination versus BCP

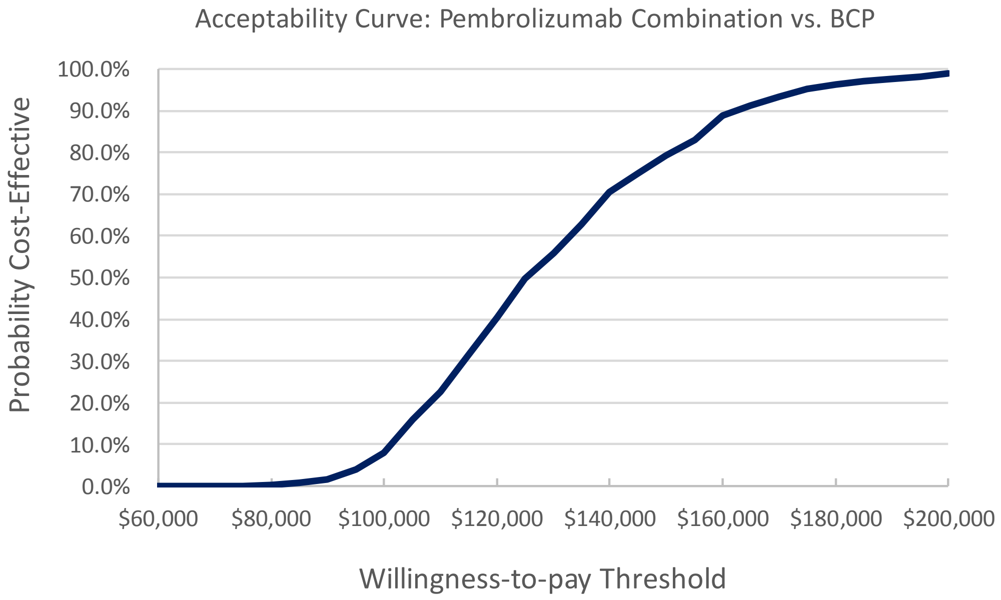


To explore the effect of uncertainty in model parameters on the outcomes, we performed a PSA with 1,000 iterations to test the robustness of our results to variations in key model inputs. Each iteration sampled the distributions of 24 different variables for health state utilities, costs, survival, body weight, and body surface area. We assumed triangular distributions for costs and survival, and normal distributions for utilities, body weight, and body surface area. Upper and lower limits for triangular distributions and 95% confidence intervals for normal distributions can be found in eTable5. Variation in different parameters were assumed to be independent.

© 2019 Criss SD et al. *JAMA Network Open.*

**eReferences**

1. Socinski MA, Jotte RM, Cappuzzo F, et al. Atezolizumab for First-Line Treatment of Metastatic Nonsquamous NSCLC. *N Engl J Med.* 2018;378(24):2288-2301.
2. Gandhi L, Rodriguez-Abreu D, Gadgeel S, et al. Pembrolizumab plus Chemotherapy in Metastatic Non-Small-Cell Lung Cancer. *N Engl J Med.* 2018.
3. Herbst RS, Baas P, Kim DW, et al. Pembrolizumab versus docetaxel for previously treated, PD-L1-positive, advanced non-small-cell lung cancer (KEYNOTE-010): a randomised controlled trial. *Lancet.* 2016;387(10027):1540-1550.
4. Long GV, Tykodi SS, Schneider JG, et al. Assessment of nivolumab exposure and clinical safety of 480 mg every 4 weeks flat-dosing schedule in patients with cancer. *Ann Oncol.* 2018;29(11):2208-2213.
5. Insinga RP, Vanness DJ, Feliciano JL, Vandormael K, Traore S, Burke T. Cost-effectiveness of pembrolizumab in combination with chemotherapy in the 1st line treatment of non-squamous NSCLC in the US. *J Med Econ.* 2018;21(12):1191-1205.
6. April 2019 ASP Drug Pricing Files. Centers for Medicare and Medicaid Services; 2019. https://www.cms.gov/Medicare/Medicare-Fee-for-Service-Part-B-Drugs/McrPartBDrugAvgSalesPrice/2019ASPFiles.html. Accessed May 3, 2019.
7. Physician Fee Schedule Search. Centers for Medicare & Medicaid Services; 2019. https://www.cms.gov/apps/physician-fee-schedule/search/search-criteria.aspx. Accessed April 2019.

© 2019 Criss SD et al. *JAMA Network Open.*
